# Trypanosoma brucei RAP1 Has Essential Functional Domains That Are Required for Different Protein Interactions

**DOI:** 10.1128/mSphere.00027-20

**Published:** 2020-02-26

**Authors:** Marjia Afrin, Hanadi Kishmiri, Ranjodh Sandhu, M. A. G. Rabbani, Bibo Li

**Affiliations:** a Center for Gene Regulation in Health and Disease, Department of Biological, Geological, and Environmental Sciences, College of Sciences and Health Professions, Cleveland State University, Cleveland, Ohio, USA; b Case Comprehensive Cancer Center, Case Western Reserve University, Cleveland, Ohio, USA; c Department of Inflammation and Immunity, Lerner Research Institute, Cleveland Clinic, Cleveland, Ohio, USA; Johns Hopkins Bloomberg School of Public Health

**Keywords:** RAP1, telomere, *Trypanosoma brucei*, protein-protein interaction, RAP1

## Abstract

RAP1 is a telomere protein that is well conserved from protozoa to mammals. It plays important roles in chromosome end protection, telomere length control, and gene expression/silencing at both telomeric and nontelomeric loci. Interaction with different partners is an important mechanism by which RAP1 executes its different functions in yeast. The RAP1 ortholog in Trypanosoma brucei is essential for variant surface glycoprotein (VSG) monoallelic expression, an important aspect of antigenic variation, where T. brucei regularly switches its major surface antigen, VSG, to evade the host immune response. Like other RAP1 orthologs, T. brucei RAP1 (*Tb*RAP1) has conserved functional domains, including BRCA1 C terminus (BRCT), Myb, MybLike, and RAP1 C terminus (RCT). To study functions of various *Tb*RAP1 domains, we established a strain in which one endogenous allele of *Tb*RAP1 is flanked by loxP repeats, enabling its conditional deletion by Cre-mediated recombination. We replaced the other *TbRAP1* allele with various mutant alleles lacking individual functional domains and examined their nuclear localization and protein interaction abilities. The N terminus, BRCT, and RCT of *Tb*RAP1 are required for normal protein levels, while the Myb and MybLike domains are essential for normal cell growth. Additionally, the Myb domain of *Tb*RAP1 is required for its interaction with T. brucei TTAGGG repeat-binding factor (*Tb*TRF), while the BRCT domain is required for its self-interaction. Furthermore, the *Tb*RAP1 MybLike domain contains a bipartite nuclear localization signal that is required for its interaction with importin α and its nuclear localization. Interestingly, RAP1’s self-interaction and the interaction between RAP1 and TRF are conserved from kinetoplastids to mammals. However, details of the interaction interfaces have changed throughout evolution.

**IMPORTANCE** Trypanosoma brucei causes human African trypanosomiasis and regularly switches its major surface antigen, VSG, to evade the host immune response. VSGs are expressed from subtelomeres in a monoallelic fashion. *Tb*RAP1, a telomere protein, is essential for cell viability and VSG monoallelic expression and suppresses VSG switching. Although *Tb*RAP1 has conserved functional domains in common with its orthologs from yeasts to mammals, the domain functions are unknown. RAP1 orthologs have pleiotropic functions, and interaction with different partners is an important means by which RAP1 executes its different roles. We have established a Cre-loxP-mediated conditional knockout system for *Tb*RAP1 and examined the roles of various functional domains in protein expression, nuclear localization, and protein-protein interactions. This system enables further studies of *Tb*RAP1 point mutation phenotypes. We have also determined functional domains of *Tb*RAP1 that are required for several different protein interactions, shedding light on the underlying mechanisms of *Tb*RAP1-mediated VSG silencing.

## Abstract (precis)

Trypanosoma brucei causes human African trypanosomiasis and regularly switches its major surface antigen, VSG, to evade the host immune response. VSGs are expressed from subtelomeres in a monoallelic fashion. *Tb*RAP1, a telomere protein, is essential for cell viability and VSG monoallelic expression and suppresses VSG switching. Although *Tb*RAP1 has conserved functional domains in common with its orthologs from yeasts to mammals, the domain functions are unknown. RAP1 orthologs have pleiotropic functions, and interaction with different partners is an important means by which RAP1 executes its different roles. We have established a Cre-loxP-mediated conditional knockout system for *Tb*RAP1 and examined the roles of various functional domains in protein expression, nuclear localization, and protein-protein interactions. This system enables further studies of *Tb*RAP1 point mutation phenotypes. We have also determined functional domains of *Tb*RAP1 that are required for several different protein interactions, shedding light on the underlying mechanisms of *Tb*RAP1-mediated VSG silencing.

## INTRODUCTION

Telomeres are nucleoprotein complexes at linear chromosome ends. They are essential for genome integrity and chromosome stability (1). Telomere DNA in most eukaryotic cells consists of TG-rich tandem repeats (2), and proteins that associate with the telomere chromatin play critical roles in all aspects of telomere biology, including telomere length regulation (3) and protection of the natural chromosome ends from nucleolytic degradation and illegitimate DNA damage repair processes (1).

Among the core telomere protein components, RAP1 orthologs have been identified in many eukaryotes, including vertebrates (4–7), yeasts (8–15), and kinetoplastids (16). RAP1 orthologs have similar functions in chromosome end protection (17–27), telomere length control (4, 11, 17, 28–37), and telomeric silencing (12, 16, 34, 38–44) — where the expression of genes located at subtelomeric regions are suppressed by the heterochromatic telomere structure (45). Strikingly, RAP1 orthologs also have nontelomeric functions, including both transcription activation and repression activities (8, 46–55). In budding yeast, Saccharomyces cerevisiae RAP1 (*Sc*RAP1) achieves different goals through interactions with various protein partners (50). For example, the C-terminal domain of *Sc*RAP1 interacts with Sir3 and Sir4, which helps maintain telomeric silencing (39, 56, 57), and the same region of *Sc*RAP1 also interacts with Rif1 and Rif2 to regulate the telomere length (58, 59). Interestingly, RAP1 orthologs have several conserved protein-protein interaction domains. All known RAP1s also have a BRCA1 C terminus (BRCT) domain (4, 11, 12, 16) that is found in many proteins involved in DNA damage repair and replication (60, 61) and that frequently interacts with phosphorylated peptides (62–64). Human and yeast RAP1 orthologs also have a C-terminal conserved domain termed RAP1 C terminus (RCT) (4, 65), which is mainly involved in protein-protein interactions (39, 56–59, 66–68). All known RAP1 domains have a central Myb domain (4, 11–13, 16, 69). Although *Sc*RAP1 uses its Myb and MybLike domains to bind duplex DNA directly (69), the human RAP1 Myb domain does not have any DNA binding activity, as its third helix has a negatively charged surface that is not suitable for DNA recognition (70).

A RAP1 ortholog has been identified in Trypanosoma brucei (16), a protozoan parasite that causes human African trypanosomiasis. T. brucei proliferates in extracellular spaces of its mammalian host and is directly exposed to the host immune surveillance. However, the parasite regularly switches its major surface antigen, variant surface glycoprotein (VSG), thereby effectively evading the host immune response (71). The T. brucei genome has >2,500 *VSG* genes and pseudogenes (72), which are all located at subtelomeres (72–74). VSGs are expressed exclusively from VSG expression sites (ESs), which are subtelomeric polycistronic transcription units transcribed by RNA polymerase I (RNA Pol I) (75, 76). *VSG* is the last gene in any ES, located within 2 kb of the telomere repeats, while the ES promoter is 40 to 60 kb upstream (73). There are 13 different ESs in the Lister 427 strain (74), all with the same gene organization and with ∼90% sequence identity (73). However, at any given moment, only one ES is fully transcribed, presenting a single type of VSG on the cell surface (77). Monoallelic VSG expression ensures the effectiveness of VSG switching by avoiding presentation of a previously active VSG on the cell surface after a VSG switch, which helps the parasite to establish long-term infections. Many factors have been shown to regulate monoallelic VSG expression, including chromatin structure, transcription elongation, inositol phosphate pathway, and nuclear lamina (78, 79); a subtelomere and VSG-associated VEX complex (80, 81); and telomeric silencing (16, 44). VSG switching has two major pathways (82, 83). In an *in situ* switch, the originally active ES is silenced while a different one becomes fully active (82, 83). In recombination-mediated switches, either a silent *VSG* gene exchanges places with the originally active *VSG* without any loss of genetic information or a silent *VSG* gene is duplicated into the active ES to replace the originally active *VSG* gene (84). Many factors important for homologous recombination, DNA damage repair, and DNA replication influence VSG switching frequencies (84). Several telomere proteins also suppress VSG switching (85–88).

T. brucei RAP1 (*Tb*RAP1) was identified as a factor interacting with T. brucei TTAGGG repeat-binding factor (*Tb*TRF) (16), which binds the duplex telomere DNA directly (89). *Tb*RAP1 is essential for cell proliferation, and depletion of *Tb*RAP1 leads to a dramatic derepression of all ES-linked *VSG* genes up to >1,000-fold (16, 44). Transient depletion of *Tb*RAP1 also results in an increased VSG switching frequency (88). Additionally, depletion of *Tb*RAP1 leads to an increased level of the telomeric transcript (TERRA), an increased amount of telomeric RNA:DNA hybrids, and an elevated amount of telomeric/subtelomeric DNA damage (88). We showed that *Tb*RAP1 has a BRCT domain located toward its N terminus, a central Myb domain, and a weak MybLike domain toward its C terminus (16). Here, we report that *Tb*RAP1 also has a C-terminal RCT domain. However, functions of *Tb*RAP1 domains are poorly understood. The interaction interface between *Tb*RAP1 and *Tb*TRF is unknown. Whether this interaction is required for the nuclear localization of *Tb*RAP1 is unclear. These limitations have hindered further investigation of how *Tb*RAP1 regulates VSG silencing and switching.

In this study, we established several strains in which one endogenous *TbRAP1* allele is flanked by two loxP repeats so that it can be conditionally deleted by inducing Cre expression. Using this system, we determined that *Tb*RAP1 Myb is necessary for *Tb*TRF interaction and VSG silencing. Using *Tb*RAP1 MybLike as bait in a yeast 2-hybrid screen, we have determined that importin α interacts with *Tb*RAP1’s nuclear localization signal (NLS) residing in the MybLike domain. We found that *Tb*RAP1 interacts with itself through the BRCT domain. In addition, the N terminus, BRCT, and RCT of *Tb*RAP1 are required for normal *Tb*RAP1 protein levels, while Myb and MybLike are essential for normal cell growth. These results not only provide further evidence of conserved essential functions of RAP1 orthologs throughout evolution but also pave the way for a better understanding of the mechanisms explaining how *Tb*RAP1 silences subtelomeric *VSG* genes and helps maintain telomere stability and integrity.

## RESULTS

### Establishing a T. brucei strain with a floxed *TbRAP1* allele.

**FIG 1:**
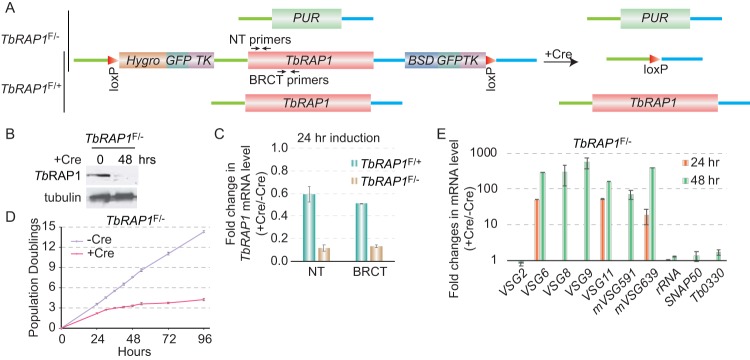
Conditional knockout of T. brucei RAP1 (*Tb*RAP1) led to cell growth arrest and VSG derepression. (A) A diagram showing three different *TbRAP1* alleles, including deleted (top), floxed (middle), and WT (bottom) alleles, in *TbRAP1*^F/−^ (top two) and *TbRAP1*^F/+^ (bottom two) cells before (left) and after (right) the Cre induction. NT, N terminus. (B) Western analysis of cell lysates prepared from *TbRAP1*^F/−^ cells before and after the Cre induction. A rabbit antibody (16) was used to detect *Tb*RAP1 (top). In this and other figures, TAT-1 (107) was used to detect tubulin (as a loading control). (C) Quantitative RT-PCR was performed using *Tb*RAP1 primers that anneal to the N terminus or the BRCT domain of *Tb*RAP1 (marked in panel A) to estimate the change in the *TbRAP1* mRNA level in *TbRAP1*^F/+^ and *TbRAP1*^F/−^ strains. (D) Growth curves of *TbRAP1*^F/−^ cells with and without the doxycycline-induced Cre expression. (E) Quantitative RT-PCR to estimate the changes in mRNA level of a number of ES-linked *VSG* genes and several control genes. Average values were calculated from three independent inductions. In this and following figures, error bars represent standard deviations.

*Tb*RAP1 is an essential protein (16), making it difficult to study phenotypes of various small deletions or point mutations using RNA interference (RNAi) (90). To better characterize functions of *Tb*RAP1, we established a strain in which one *TbRAP1* allele was flanked by two loxP repeats so that it was able to be conditionally deleted through Cre-mediated recombination (Fig. 1A). The other *TbRAP1* allele can be replaced by various *Tb*RAP1 mutants. Upon induction of Cre by the use of doxycycline, we are able to examine the phenotypes of the mutant *Tb*RAP1, even if they are lethal.

A loxP site with the hygromycin resistance gene (*HYG*) and a loxP site with the blasticidin resistance gene (*BSD*) were targeted to locations upstream and downstream, respectively, of a given *TbRAP1* allele (Fig. 1A). Both selectable markers were fused with the thymidine kinase gene (*TK*) from the herpes simplex virus, allowing selection for cells that had lost the floxed *TbRAP1* allele (denoted as “F”) by the use of ganciclovir (GCV), as expression of the *TK* gene in T. brucei renders the parasite sensitive to GCV (91). To enable selection of cells that had lost the floxed allele by the use of fluorescence-activated cell sorting (FACS) analysis, we also fused the green fluorescent protein gene (*GFP*) with both selectable markers (Fig. 1A). Therefore, cells carrying a floxed *TbRAP1* allele expressed HYG-GFP-TK and BSD-GFP-TK fusion proteins, and this was confirmed by Western analysis using a rabbit anti-GFP antibody (Life Technologies) in two *Tb*RAP1^F/+^ clones (without the Cre expression construct Cre-EP1 [91]) (see Fig. S1A in the supplemental material). Southern blotting also confirmed the genotype of these clones (Fig. S1B).

10.1128/mSphere.00027-20.1 FIG S1 Validation of Cre-loxP-mediated conditional deletion of *Tb*RAP1. (A) Western blotting using a rabbit GFP antibody (Life Technologies) to detect HYG-GFP-TK and BSD-GFP-TK expression in *TbRAP1*^F/+^ (-Cre-Ep1) cells before and after transient transfection of Cre-EP1 (91). (B) Southern blotting of BamHI/XbaI-digested genomic DNA isolated from *TbRAP1*^F/+^ (-Cre-Ep1) cells before and after transient transfection of Cre-EP1 (91). A DNA fragment containing the full-length *TbRAP1* gene was used as the probe. The WT *TbRAP1* allele gave a 7.9-kb band, while the floxed *TbRAP1* allele gave a 4.9-kb band. (C) Growth curves of two clones of *TbRAP1*^F/+^ cells (with Cre-EP1 targeted to an rDNA spacer region) before and after induction of Cre by doxycycline. Cells were selected with different combinations of antibiotics as indicated. The Cre-EP1 plasmid has a phleomycin resistance (*BLE*) marker (91). Download FIG S1, EPS file, 1.9 MB. Copyright © 2020 Afrin et al. 2020 Afrin et al. This content is distributed under the terms of the Creative Commons Attribution 4.0 International license.

To validate that the floxed *TbRAP1* allele can be excised by Cre efficiently, we transiently transfected the Cre-EP1 plasmid into *TbRAP1*^F/+^ (-Cre-EP1) cells. Pools of cells and individual clones were selected with GCV. Western analysis performed with the GFP antibody showed that HYG-GFP-TK and BSD-GFP-TK were no longer expressed in either the pool or the clones (Fig. S1A). Southern analysis further confirmed that the floxed *TbRAP1* allele was lost in the selected pool and clones (Fig. S1B).

To further increase the feasibility of conditional deletion of the floxed *TbRAP1* allele, we integrated Cre-EP1 into a ribosomal DNA (rDNA) spacer region, whose expression can be induced by doxycycline (91). The Cre-EP1-integrated *TbRAP1*^F/+^ cells grew normally in the presence of phleomycin, hygromycin, and blasticidin (Fig. S1C). Upon addition of doxycycline, these cells still grew normally in the presence of phleomycin only but did not survive in the presence of all three antibiotics (phleomycin, hygromycin, and blasticidin) (Fig. S1C), confirming that the doxycycline-induced Cre had excised the floxed *TbRAP1* allele together with the *HYG-GFP-TK* and *BSD-GFP-TK* markers. In the subsequent studies, the Cre-EP1 integrated *TbRAP1*^F/+^ strain was used.

### Conditional deletion of *Tb*RAP1 leads to cell growth arrest and VSG derepression.

In *TbRAP1*^F/+^ cells, we replaced the unfloxed *TbRAP1* allele with a puromycin resistance marker (*PUR*) (Fig. 1A). In the resulting *TbRAP1*^F/−^ cells, addition of doxycycline led to the loss of the *Tb*RAP1 protein in 48 h (Fig. 1B), confirming the efficient deletion of the floxed *TbRAP1* allele. The *Tb*RAP1 mRNA level was estimated by quantitative reverse transcription PCR (RT-PCR), and two sets of *Tb*RAP1 primers were used: one annealed specifically to the N-terminal region and another annealed to the BRCT domain (Fig. 1A). After 24 h of Cre induction, the *Tb*RAP1 mRNA level dropped to 10% of the wild-type (WT) level in *TbRAP1*^F/−^ cells (Fig. 1C), indicating that the sole *TbRAP1* allele in these cells was excised by Cre. In contrast, the *Tb*RAP1 mRNA level dropped to ∼50% of the WT level in *TbRAP1*^F/+^ cells (Fig. 1C), as only the floxed *TbRAP1* allele had been deleted, leaving the other WT allele intact.

We previously showed that depletion of *Tb*RAP1 by RNAi led to cell growth arrest and VSG derepression (16, 44). Induction of Cre in *TbRAP1*^F/−^ cells also led to a severe growth defect (Fig. 1D). Quantitative RT-PCR analysis showed that ES-linked *VSG6*, *VSG8*, *VSG9*, *VSG11*, *VSG591*, and *VSG639* (all originally silent) were derepressed by several 10-fold orders after induction of Cre for 24 h and by several 100-fold orders after 48 h (Fig. 1E). As a control, the rRNA levels and mRNA levels of two chromosome-internal genes, *SNAP50* and *Tb11.0330*, did not increase significantly (Fig. 1E). The mRNA level of the originally active *VSG2* gene was decreased ∼20% (Fig. 1E). Therefore, conditional deletion of *Tb*RAP1 by Cre-loxP exhibited the same phenotypes as depletion of *Tb*RAP1 by RNAi (16), indicating that the Cre-loxP-mediated conditional deletion is feasible and efficient.

### The *Tb*RAP1 Myb domain is essential for normal cell growth and VSG silencing.

**FIG 2:**
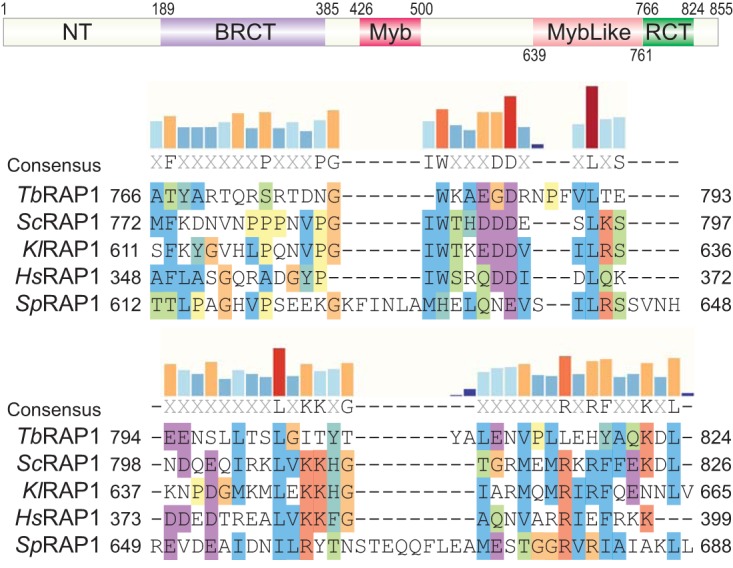
*Tb*RAP1 has an RCT domain. (Top) *Tb*RAP1 domain structure. The positions of each domain are labeled. NT, N terminus; BRCT, BRCA1 C terminus; RCT, RAP1 C terminus. (Bottom) Sequence alignment of the *Tb*RAP1 RCT domain and the RCT domains in Saccharomyces cerevisiae (*Sc*) RAP1, Kluyveromyces lactis (*Kl*) RAP1, Homo sapiens (*Hs*) RAP1, and Schizosaccharomyces pombe (*Sp*) RAP1 performed using Clustal X. Alignment is visualized in SnapGene. Amino acid positions are indicated. Amino acids are highlighted based on their properties and level of conservation (Clustal X). Specifically, nonpolar amino acids are highlighted in blue, positively charged amino acids in red, negatively charged amino acids in magenta, G amino acid (lacking of bonding characteristics) in orange, H and Y amino acids (mixture of nonpolar and polar ends to the side chain) in dark turquoise, P amino acid (between polar and nonpolar) in yellow, and S and T amino acids (mostly nonpolar) in green. Levels of conservation at each position (percent identical residues among the five RAP1 orthologs) are shown as bar graphs on top of the aligned sequences.

We previously showed that *Tb*RAP1 has a BRCT domain, a Myb domain, and a MybLike domain (Fig. 2, top) (16). With a careful sequence analysis, we found that the C terminus of *Tb*RAP1 has recognizable similarities to the RCT domains of other RAP1 orthologs (Fig. 2, bottom). The level of sequence identity between *Tb*RAP1 and other RAP1 orthologs in this domain was 11.7%, which is approximately the same as that in the BRCT domain (16). We named this region “RCT.” Therefore, *Tb*RAP1 has several conserved domains like other known RAP1 orthologs (Fig. 2, top).

**FIG 3:**
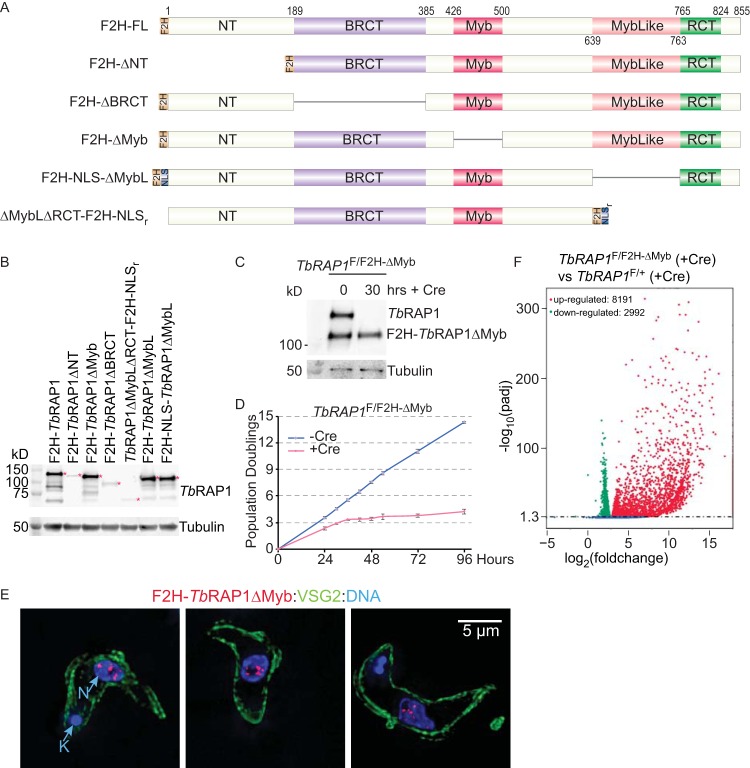
The Myb domain of *Tb*RAP1 is essential for normal cell growth and VSG silencing. (A) Diagrams of FLAG-HA-HA (F2H)-tagged WT and mutant *Tb*RAP1 used in this study. NLS, simian virus 40 (SV40) large T nuclear localization signal. NLS_r_, the second half of *Tb*RAP1 nuclear localization signal (aa 737 to 742). (B) Whole-cell lysates from *Tb*RAP1^F/F2H-mut^ cells (after induction of Cre for 30 h) and *Tb*RAP1^−/F2H+^ cells (as a positive control) were analyzed by Western blotting. HA probe monoclonal antibody (Santa Cruz Biotechnologies) was used to detect the F2H-tagged *Tb*RAP1. Red asterisks indicate the *Tb*RAP1 fragments. F2H-*Tb*RAP1ΔNT ran at a much lower rate than expected. (C) Western blotting of cell lysates prepared from *TbRAP1*^F/F2H-ΔMyb^ cells before and after induction of Cre. An anti-*Tb*RAP1 rabbit antibody (16) was used to detect *Tb*RAP1 and F2H-*Tb*RAP1ΔMyb. To differentiate WT and mutant *Tb*RAP1, proteins were separated on a 7.5% Tris polyacrylamide gel for 7 h 40 min. (D) Growth curves of *TbRAP1*^F/F2H-ΔMyb^ cells with and without the Cre induction. (E) F2H-*Tb*RAP1ΔMyb is located in the nucleus. The monoclonal HA antibody 12CA5 (Memorial Sloan Kettering Cancer Center [MSKCC] monoclonal antibody core) was used to detect F2H-*Tb*RAP1ΔMyb. An anti-VSG2 rabbit antibody (16) was used to show the outline of the cell body. DAPI was used to stain DNA. The small DAPI-positive circle represents the kinetoplast (K), and the large DAPI-positive circle represents the nucleus (N). Three different cells are shown in three panels. In this and other figures, IF images are in the same scale and a size bar is shown in one of the panels. (F) Differential gene expression in the *Tb*RAP1ΔMyb mutant was summarized in a volcano plot. *TbRAP1*^F/F2H-ΔMyb^ and *TbRAP1*^F/+^ cells were induced for Cre expression for 30 h and analyzed by RNA-seq. Compared to *TbRAP1*^F/+^ cells, more than 8,000 genes were upregulated and nearly 3,000 genes were downregulated in the Cre-induced *TbRAP1*^F/F2H-ΔMyb^ cells. A log_10_(adjusted *P* [padj]) value of 1.3 or higher is considered to be significant.

The *TbRAP1*^F/+^ strain would be a good choice to examine phenotypes of various *Tb*RAP1 mutants lacking individual domains. As a proof of principle, in the *TbRAP1*^F/+^ strain, we replaced the WT *TbRAP1* allele with a mutant that lacks the Myb domain. Because the C-terminal FLAG-hemagglutinin-hemagglutinin (FLAG-HA-HA [F2H])-tagged *Tb*RAP1ΔMyb did not express well, we replaced the WT *TbRAP1* allele with a *TbRAP1*^F2H-ΔMyb^ mutant (Fig. 3A). Western analysis using the HA probe antibody (Santa Cruz Biotechnologies) showed that F2H-*Tb*RAP1ΔMyb was expressed at the same level as F2H-*Tb*RAP1 from *TbRAP1*^−/F2H+^ cells (Fig. 3B; see also Table S1 in the supplemental material). Southern blotting confirmed the genotype of *TbRAP1*^F/F2H-ΔMyb^ (Fig. S2A). Using a *Tb*RAP1 rabbit antibody (16) that recognizes a recombinant *Tb*RAP1 fragment containing only the MybLike domain (Fig. S2B), both WT *Tb*RAP1 and F2H-*Tb*RAP1ΔMyb were observed by Western analysis at the same level in *TbRAP1*^F/F2H-ΔMyb^ cells (Fig. 3C). Upon induction of Cre, WT *Tb*RAP1 was depleted whereas the expression of F2H-*Tb*RAP1ΔMyb remained the same (Fig. 3C). F2H-*Tb*RAP1ΔMyb did not support normal cell growth as *TbRAP1*^F/F2H-ΔMyb^ cells exhibited a severe growth defect after Cre induction (Fig. 3D), even though the mutant is located in the nucleus in immunofluorescence (IF) analysis (Fig. 3E).

10.1128/mSphere.00027-20.2 FIG S2 (A) Southern analysis confirmed the *TbRAP1*^F/F2H-ΔMyb^ genotype. Genomic DNA from *TbRAP1*^F/+^ and a number of candidates of the *TbRAP1*^F/F2H-ΔMyb^ strain was digested with EcoRI and BamHI and probed with a DNA fragment containing the region of the *TbRAP1* gene before the BRCT domain. The three different *TbRAP1* alleles are labeled. C1, B1, and B3 were positive clones, while the other three did not have the intended genotype. (B) The rabbit *Tb*RAP1 antibody (16) used in this study recognizes an epitope(s) inside the MybLike domain. (Left) Polyacrylamide gel electrophoresis of the partially purified TRX-His_6_-*Tb*RAP1-MybLike fragment. (Right) Western blotting of the same recombinant fragment using the rabbit *Tb*RAP1 antibody. (C and D) Classification of genes affected in Cre-induced *TbRAP1*^F/F2H-ΔMyb^ cells. Distributions (percent of total affected genes) of different types of genes whose mRNA levels were upregulated (C) or downregulated (D) are shown. The number of genes affected in each category is listed in parentheses. Download FIG S2, EPS file, 2.7 MB. Copyright © 2020 Afrin et al. 2020 Afrin et al. This content is distributed under the terms of the Creative Commons Attribution 4.0 International license.

10.1128/mSphere.00027-20.7 TABLE S1 T. brucei strains used in the current study. Download Table S1, DOCX file, 0.01 MB. Copyright © 2020 Afrin et al. 2020 Afrin et al. This content is distributed under the terms of the Creative Commons Attribution 4.0 International license.

*Sc*RAP1 has both transcription activation and repression functions (8, 50). To examine whether the Myb domain is required for VSG silencing and affects expression of other genes, we performed transcriptome sequencing (RNA-seq) analysis. Both *TbRAP1*^F/+^ and *TbRAP1*^F/F2H-ΔMyb^ cells were induced for Cre expression for 30 h, after which the total RNA was isolated. Poly(A) RNA was purified and used for library construction at Novogene followed by paired-end high-throughput sequencing using Illumina (Materials and Methods). Differential gene expression analysis showed that more than 8,000 genes were upregulated and nearly 3,000 genes were downregulated in Cre-induced *TbRAP1*^F/F2H-ΔMyb^ cells compared to the results seen with *TbRAP1*^F/+^ (Fig. 3F). However, the fold change in mRNA levels was much greater for upregulated genes than for downregulated ones (Fig. 3F), and upregulated genes were present in much greater numbers than downregulated ones, suggesting that *Tb*RAP1 has a major role in gene silencing and a minor role in gene activation. Sequence read coverage in all VSG bloodstream-form (BF) expression sites (BESs) (74) showed that all silent BES-linked *VSG* genes and some BES-linked expression site-associated genes (*ESAG* genes) were upregulated, but other BES-linked *ESAG* genes were not affected or were even downregulated (Fig. S3). Based on available annotation of the affected genes, a total of more than 2,700 *VSG* genes and pseudogenes were upregulated (Fig. S2C), which included nearly all reported *VSG* genes/pseudogenes in the Lister 427 genome (72). Therefore, the *Tb*RAP1 Myb domain is essential for the functions of *Tb*RAP1 in normal cell growth and VSG silencing. Interestingly, the mRNA levels of some ribosomal protein genes were decreased in the mutant, although at only up to 60% of the normal level (Fig. S2D). It is possible that *Tb*RAP1 may also participate in transcription activation of ribosomal protein genes, as was seen previously with *Sc*RAP1 (8), although further investigation is necessary to validate this.

10.1128/mSphere.00027-20.3 FIG S3 RNA sequence read coverage in all VSG bloodstream-form expression sites (BESs) for Cre-induced *TbRAP1*^F/+^ and *TbRAP1*^F/F2H-ΔMyb^ cells. RNA samples were isolated from three independently induced cells in both strains. RNA sequence reads were mapped to the T. brucei Lister 427 genome TriTrypDB-45_TbruceiLister427_2018_Genome.fasta, which was obtained from TriTrypDB. Read coverage for each sample was viewed in IGV. Each BES is labeled, and the genes inside each BES are listed at the bottom. The whole BES region is shown with the promoter located on the left side. The *y* axis data represent the number of RNA reads. The range is indicated on the left of each trace. BES1 was fully active in the cells analyzed. Download FIG S3, PDF file, 1.2 MB. Copyright © 2020 Afrin et al. 2020 Afrin et al. This content is distributed under the terms of the Creative Commons Attribution 4.0 International license.

### The nuclear localization signal of *Tb*RAP1 is required for its interaction with Importin α and nuclear localization.

**FIG 4:**
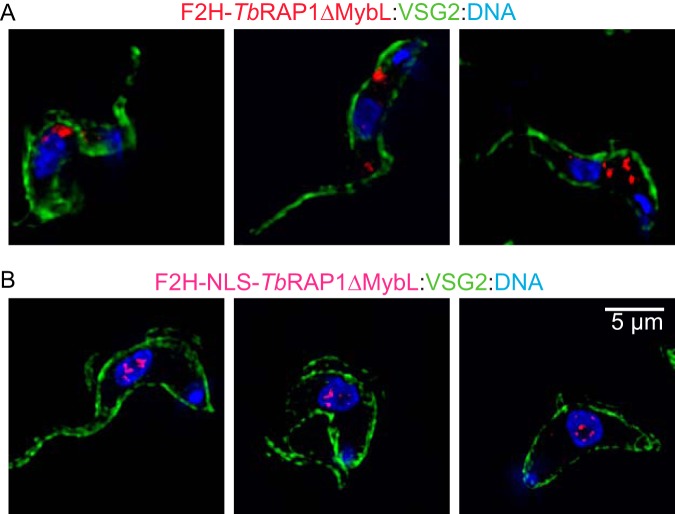
Immunofluorescent analysis of *TbRAP1*^F/F2H-ΔMybL^ TRFi and *TbRAP1*^F/F2H-NLS-ΔMybL^ TRFi cells. F2H-*Tb*RAP1ΔMybL and F2H-NLS-*Tb*RAP1ΔMybL were stained with 12CA5 HA antibody. A rabbit VSG2 antibody was used to show the outline of the cell body. DNA was stained with DAPI. In each strain, three different cells are shown in three panels.

Using the same approach, we replaced the WT *TbRAP1* allele with an F2H-tagged *Tb*RAP1 mutant lacking the MybLike domain (*Tb*RAP1ΔMybL). Southern blotting confirmed its genotype (Fig. S4A). Western blotting showed that F2H-*Tb*RAP1ΔMybL was expressed (Fig. S4B) and that the expression was at the same level as F2H-*Tb*RAP1 (Fig. 3B). However, IF showed that this mutant was localized in the cytoplasm (Fig. 4A). Using Motif Scan analysis (https://myhits.isb-sib.ch/cgi-bin/motif_scan) (92), we found that the sequence consisting of amino acids (aa) 727 to 741 of *Tb*RAP1 represents a bipartite nuclear localization signal (NLS). F2H-*Tb*RAP1ΔMybL lacks this NLS, which is likely why this mutant is localized in the cytoplasm. To confirm this, we added the simian virus 40 (SV40) large T NLS to the N terminus of *Tb*RAP1ΔMybL (Fig. 3A). Southern and Western analyses confirmed the genotype of this strain and that the expression of F2H-NLS-*Tb*RAP1ΔMybL was at the WT level (Fig. 3B; see also Fig. S4C and D). IF analysis showed that F2H-NLS-*Tb*RAP1ΔMybL was indeed localized in the nucleus (Fig. 4B).

10.1128/mSphere.00027-20.4 FIG S4 Southern analyses confirmed the genotypes of *TbRAP1*^F/F2H-ΔMybL^ (A) and *TbRAP1*^F/F2H-NLS-ΔMybL^ (C) cells. Genomic DNA was digested with EcoRI and probed with a DNA fragment amplified by PCR with the forward primer (5′-GCGCCTCGAGACCGACGTTGACGGTG-3′) and the backward primer (5′-GCGCTCTAGAACACGAAGAAATCGGGACGTG-3′) using *TbRAP1* as the template. Western analyses showed the expression levels of F2H-*Tb*RAP1ΔMybL (B) and F2H-NLS-*Tb*RAP1ΔMybL (D) in the same clones as those indicated in panels A and C, respectively. Download FIG S4, EPS file, 1.1 MB. Copyright © 2020 Afrin et al. 2020 Afrin et al. This content is distributed under the terms of the Creative Commons Attribution 4.0 International license.

To further explore how *Tb*RAP1 is imported into the nucleus, we screened a normalized yeast 2-hybrid library generated from T. brucei cDNA using the *Tb*RAP1 MybLike domain as bait. In this screen, 16.5 million yeast primary transformants were obtained, and a total of 711 clones were positive in the initial screen. The majority of the candidates represented the same gene, *Tb*427.06.2640, which is annotated as encoding the importin α subunit in TriTrypDB (93, 94). The canonical function of importin α is to bind the NLS of nuclear proteins, form a complex with importin β, and transport the protein into the nucleus through the nuclear pore (95). Once inside the nucleus, importin α releases its cargo and exits the nucleus to transport the next cargo (95). *Tb*RAP1 MybLike contains the predicted bipartite NLS. Therefore, we expected that importin α would interact with the *Tb*RAP1 NLS and this interaction would be essential for transporting *Tb*RAP1 into the nucleus.

**FIG 5:**
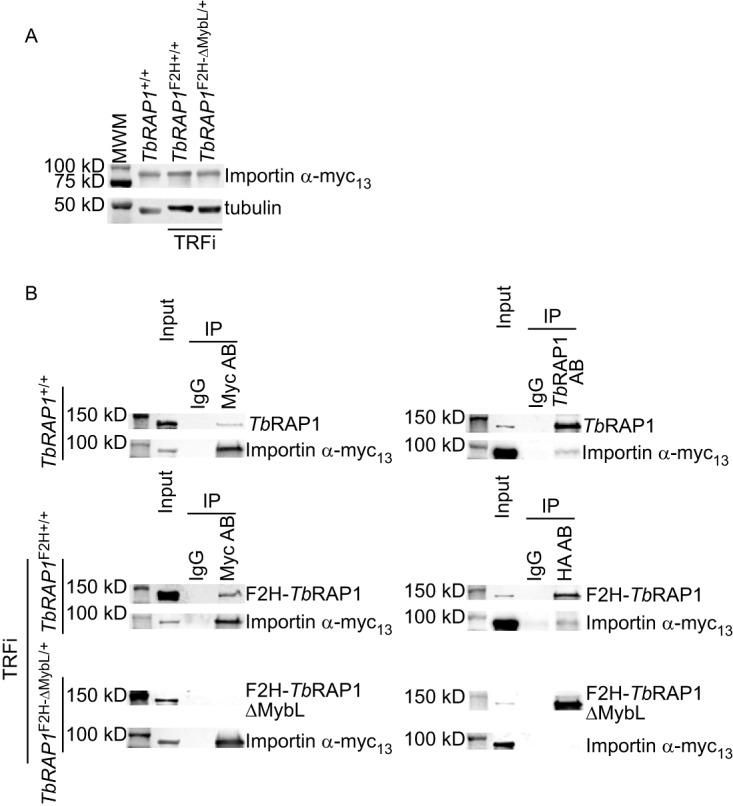
The *Tb*RAP1 MybLike domain is required for interaction with importin α. (A) Expression of importin α-myc_13_ in *TbRAP1*^+/+^, *TbRAP1*^F2H+/+^ TRFi, and *TbRAP1*^F2H-ΔMybL/+^ TRFi cells. Myc_13_-tagged proteins were detected by a myc monoclonal antibody, 9E10 (MSKCC monoclonal antibody core). (B) Co-IP of *Tb*RAP1 and importin α. The myc antibody 9E10, an anti-*Tb*RAP1 rabbit antibody (16), and IgG (as a negative control) were used for IP in *TbRAP1*^+/+^ cells. Western analysis was performed using the antibodies mentioned above to detect importin α-myc_13_ and *Tb*RAP1. In *TbRAP1*^F2H+/+^ TRFi and *TbRAP1*^F2H-ΔMybL/+^ TRFi cells, the 9E10 myc antibody, the 12CA5 HA antibody, and IgG (as a negative control) were used for IP, and Western blotting was performed to detect importin α-myc_13_ (by 9E10) and F2H-tagged WT and mutant *Tb*RAP1 (by 12CA5 in the left panels and HA probe in the right panels). In this and other figures, input samples represent 1% of the materials used for IP.

To confirm the interaction between importin α and *Tb*RAP1, we inserted a C-terminal myc_13_ (13 repeats of myc) epitope at one endogenous importin α allele. PCR analysis confirmed correct targeting in three different *TbRAP1* backgrounds: both alleles were WT, one of the two WT alleles had an N-terminal F2H tag, and one of the alleles was replaced with the *TbRAP1*^F2H-ΔMybL^ mutant (Fig. S5). The latter two strains also carried an inducible *Tb*TRF RNAi cassette inserted into an rDNA spacer, although the RNAi was not induced for the analysis of *Tb*RAP1-importin α interaction. The expression of importin α-myc_13_ was confirmed by Western blotting (Fig. 5A). Subsequently, we performed coimmunoprecipitation (co-IP) experiments in these three strains. In the *TbRAP1*^+/+^ background, IP experiments were performed using a rabbit *Tb*RAP1 antibody (16) or the myc monoclonal antibody 9E10 (Memorial Sloan Kettering Cancer Center [MSKCC] monoclonal antibody core). In *TbRAP1*^F2H+/+^ TRF RNAi (TRFi) and *TbRAP1*^F2H-ΔMybL/+^ TRFi cells, IP experiments were performed using HA monoclonal antibody 12CA5 (MSKCC monoclonal antibody core) or 9E10. In cells carrying untagged or F2H-tagged WT *Tb*RAP1, both importin α-myc_13_ and *Tb*RAP1 were present in the IP products (Fig. 5B, top two rows). However, F2H-*Tb*RAP1ΔMybL and importin α-myc_13_ were not in the same IP product (Fig. 5B, bottom row). Therefore, the MybLike domain of *Tb*RAP1 (and most likely the NLS in this domain) is necessary for *Tb*RAP1’s interaction with importin α and its nuclear localization.

10.1128/mSphere.00027-20.5 FIG S5 PCR confirmed the endogenous tagging of importin α with a C-terminal myc_13_ tag. Genomic DNAs from the parent *TbRAP1*^+/+^ strain (as a negative control) and three transfected cells in the *TbRAP1*^+/+^, *TbRAP1*^F2H+/+^ TRFi, and *TbRAP1*^F2H-ΔMybL/+^ TRFi background were used as the templates. Forward primer (5′-CACTTTGTCTCCTCGAGACA-3′) and backward primer (5′-CGATCAGAAACTTCTCGACAG-3′) were used in the PCR amplification. A product of 1.26 kb is expected for correct targeting of the myc_13_ tag. Download FIG S5, EPS file, 1.2 MB. Copyright © 2020 Afrin et al. 2020 Afrin et al. This content is distributed under the terms of the Creative Commons Attribution 4.0 International license.

### The *Tb*RAP1 Myb domain interacts with *Tb*TRF.

**FIG 6:**
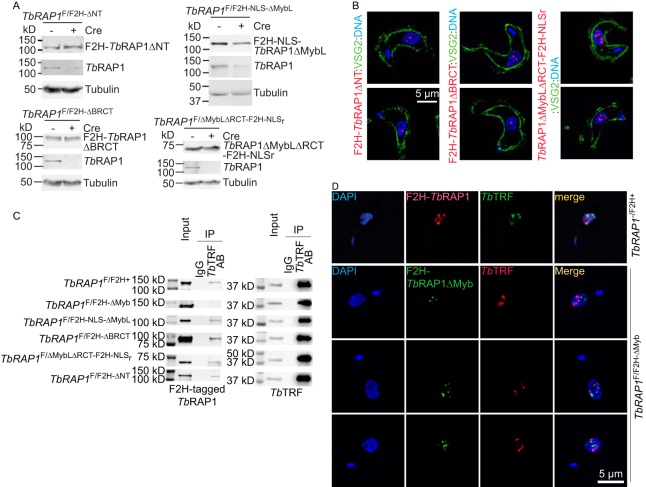
The *Tb*RAP1 Myb domain is required for interaction with *Tb*TRF. (A) Western analyses showed the expression levels of the F2H-tagged *Tb*RAP1 mutants (using the HA probe antibody) and WT *Tb*RAP1 (using a rabbit *Tb*RAP1 antibody [16]) before and after the Cre induction in *TbRAP1*^F/F2H-ΔNT^, *TbRAP1*^F/F2H-ΔBRCT^, *TbRAP1*^F/F2H-NLS-ΔMybL^, and *TbRAP1*^F/ΔMybLΔRCT-F2H-NLSr^ cells. (B) IF analysis showed that F2H-*Tb*RAP1ΔNT, F2H-*Tb*RAP1ΔBRCT, and *Tb*RAP1ΔMybLΔRCT-F2H-NLS_r_ were localized in the nucleus. 12CA5 was used to detect the F2H-tagged *Tb*RAP1 mutants. A rabbit anti-VSG2 antibody was used to show the outline of the cell body. DNA was stained by DAPI. In each strain, two different cells are shown in two panels. (C) Co-IP experiments were performed in a series of *TbRAP1*^F/F2H-mut^ cells (as labeled on the left). An anti-*Tb*TRF rabbit antibody (89) and IgG (as a negative control) were used for IP. The antibodies used for Western analyses of the IP products were the 12CA5 HA antibody (except for *TbRAP1*^F/F2H-ΔNT^, where the HA probe was used) and an anti-*Tb*TRF chicken antibody (16). (D) (Top) F2H-*Tb*RAP1 (detected by 12CA5) partially colocalized with *Tb*TRF (detected by a rabbit *Tb*TRF antibody [89]) in *TbRAP1*^−/F2H+^ cells. (Bottom) F2H-*Tb*RAP1ΔMyb (detected by 12CA5) was not colocalized with *Tb*TRF (detected by a chicken *Tb*TRF antibody [16]). DNA was stained by DAPI. Three different *Tb*RAP1^F/F2H-∆Myb^ cells are shown.

*Tb*RAP1 was originally identified as a *Tb*TRF-interacting factor in a yeast 2-hybrid screen (16). The N-terminal part of *Tb*RAP1 (including the N terminus, BRCT, and Myb) is sufficient to interact with *Tb*TRF in yeast 2-hybrid analysis (16), and WT *Tb*RAP1 and *Tb*TRF co-IP *in vivo* (16), although both assays showed a weak interaction between the two proteins. To examine which *Tb*RAP1 functional domain(s) is essential for the *Tb*RAP1-*Tb*TRF interaction, in the *TbRAP1*^F/+^ cells, we either targeted an F2H tag to the N terminus of the WT *TbRAP1* allele (to generate the *TbRAP1*^F/F2H+^ strain) or replaced the WT allele with an F2H-tagged *Tb*RAP1 mutant lacking various functional domains (to generate the *TbRAP1*^F/F2H-mut^ strains) (Fig. 3A). Southern analyses confirmed the replacement of the WT *TbRAP1* allele by the *TbRAP1*^F2H-ΔNT^, *TbRAP1*^F2H-ΔBRCT^, or *TbRAP1*^ΔMybLΔRCT-F2H-NLSr^ allele in the corresponding strains (Fig. S6A to C). Here, we added the second half (aa 736 to 742) of the endogenous *Tb*RAP1 NLS (labeled as NLS_r_) at the C terminus of *Tb*RAP1ΔMybLΔRCT. These *Tb*RAP1 mutants were expressed (Fig. 6A) but at much lower levels than F2H-*Tb*RAP1 (Fig. 3B). F2H-*Tb*RAP1ΔNT and F2H-*Tb*RAP1ΔBRCT still contained the *Tb*RAP1 NLS, and they were localized in the nucleus as expected (Fig. 6B, left and middle). Interestingly, *Tb*RAP1ΔMybLΔRCT-F2H-NLS_r_ was also localized in the nucleus (Fig. 6B, right), indicating that the sequence consisting of aa 736 to 742 contains a minimum nuclear localization signal and that its presence is sufficient to target *Tb*RAP1 to the nucleus. This observation further validated the function of *Tb*RAP1 NLS.

10.1128/mSphere.00027-20.6 FIG S6 The *TbRAP1*^F/F2H-ΔNT^, *TbRAP1*^F/F2H-ΔBRCT^, *TbRAP1*^F/F2H-NLS-ΔMybL^, and *TbRAP1*^F/ΔMybLΔRCT-F2H-NLSr^ cells exhibited a growth arrest phenotype after induction of Cre. (A to C) Southern analyses confirmed the genotype of *TbRAP1*^F/F2H-ΔNT^ (A), *TbRAP1*^F/F2H-ΔBRCT^ (B), and *TbRAP1*^F/ΔMybLΔRCT-F2H-NLSr^ (C) cells. Genomic DNAs were digested with XbaI and BamHI in the experiments represented in panels A and C and with EcoRI in the experiments represented in panel B. A DNA fragment containing the *TbRAP1* sequence between the BRCT domain and the Myb domain was used as the probe in the experiments represented in panels A and C. A DNA fragment containing the N-terminal region of *TbRAP1* was used as the probe used in the experiments represented in panel B. (D to G) Growth curves of *TbRAP1*^F/F2H-NLS-ΔMybL^ (D), *TbRAP1*^F/F2H-ΔNT^ (E), *TbRAP1*^F/F2H-ΔBRCT^ (F), and *TbRAP1*^F/ΔMybLΔRCT-F2H-NLSr^ (G) cells before and after the induction of Cre expression are shown. Download FIG S6, EPS file, 1.9 MB. Copyright © 2020 Afrin et al. 2020 Afrin et al. This content is distributed under the terms of the Creative Commons Attribution 4.0 International license.

Since human RAP1 interacts with itself (4), it is possible that *Tb*RAP1 may also interact with itself (see below). To avoid detecting possible indirect interactions between *Tb*TRF and mutant *Tb*RAP1 mediated by the WT *Tb*RAP1, we induced Cre in these *TbRAP1*^F/F2H-mut^ strains for 30 h to ensure the depletion of the WT *Tb*RAP1 protein (Fig. 6A). Depletion of *Tb*RAP1 by RNAi for up to 36 h still allows complete cell growth recovery 24 h after removal of the RNAi induction (88). In addition, upon Cre induction, the number of *TbRAP1*^F/−^ and *TbRAP1*^F/F2H-mut^ cells did not decrease for several days (Fig. 1D) (Fig. 3D; see also Fig. S6D to G). Therefore, inducing *TbRAP1*^F/F2H-mut^ for 30 h caused only cell growth arrest rather than cell death. Subsequently, co-IPs were performed using a rabbit *Tb*TRF antibody (89). In all cases, *Tb*TRF was detected in the IP products by Western analysis using a chicken *Tb*TRF antibody (16) (Fig. 6C, right). Although F2H-*Tb*RAP1ΔNT, F2H-*Tb*RAP1ΔBRCT, and *Tb*RAP1ΔMybLΔRCT-F2H-NLS_r_ were expressed at very low levels (Fig. 3B), these mutants still interacted with *Tb*TRF, as they were detected in the IP products in the same manner as F2H-*Tb*RAP1 and F2H-NLS-*Tb*RAP1ΔMybL (Fig. 6C, left). However, F2H-*Tb*RAP1ΔMyb was not detected in the IP product (Fig. 6C, left). In addition, IF analysis showed that F2H-*Tb*RAP1ΔMyb was not colocalized with *Tb*TRF even though these proteins were in the nucleus (Fig. 6D). As a control, WT *Tb*RAP1 was partially colocalized with *Tb*TRF (Fig. 6D), as we have shown previously (16). Therefore, the Myb domain of *Tb*RAP1 is required for its interaction with *Tb*TRF. Interestingly, *TbRAP1*^F/F2H-NLS-ΔMybL^ cells exhibited a growth arrest phenotype after induction of Cre for 30 h (Fig. S6D), even though F2H-NLS-*Tb*RAP1ΔMybL was expressed at the WT level (Fig. 3B) and was localized in the nucleus (Fig. 4B), indicating that the MybLike domain has essential functions other than transporting *Tb*RAP1 into the nucleus. On the other hand, F2H-*Tb*RAP1ΔNT, F2H-*Tb*RAP1ΔBRCT, and *Tb*RAP1ΔMybLΔRCT-F2H-NLS_r_ were expressed at much lower levels than WT *Tb*RAP1 (Fig. 3B), indicating that the N terminus and BRCT and RCT domain of *Tb*RAP1 are required for normal *Tb*RAP1 protein levels, which is likely the reason why *TbRAP1*^F/F2H-ΔNT^, *TbRAP1*^F/F2H-ΔBRCT^, and *TbRAP1*^F/ΔMybLΔRCT-F2H-NLSr^ cells also showed a growth arrest phenotype after induction of Cre (Fig. S6E to G).

### The BRCT domain is required for *Tb*RAP1 self-interaction.

**FIG 7:**
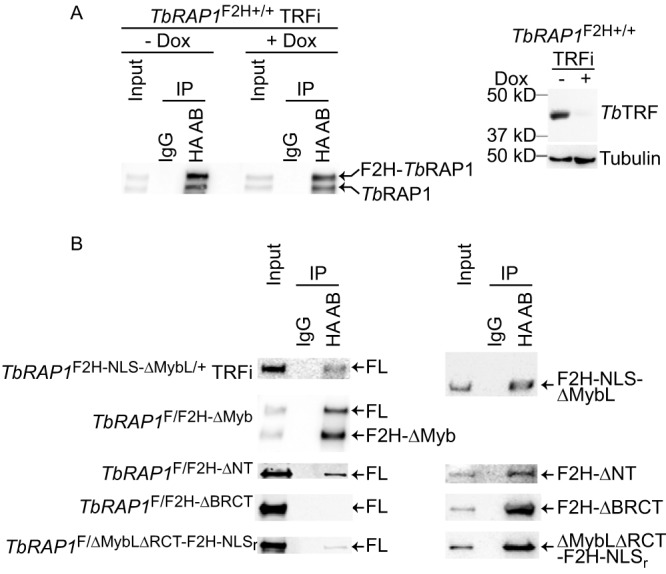
*Tb*RAP1 interacts with itself through the BRCT domain. (A) *Tb*RAP1’s self-interaction activity is independent of *Tb*TRF. Left, co-IP experiments were done using the 12CA5 HA antibody and IgG (as a negative control) in *TbRAP1*^F2H+/+^ TRFi cells before and after induction of *Tb*TRF RNAi. IP products were analyzed by Western blotting using a rabbit *Tb*RAP1 antibody (16). To differentiate F2H-*Tb*RAP1 and the untagged *Tb*RAP1, proteins were separated on a 7.5% Tris polyacrylamide gel for 7 h. Right, Western analysis showed that *Tb*TRF was efficiently depleted by RNAi after addition of doxycycline (Dox) for 30 h. A rabbit antibody was used to detect *Tb*TRF (89). (B) The BRCT domain of *Tb*RAP1 is critical for its self-interaction. Co-IP experiments were performed in various *TbRAP1*^F/F2H-mut^ strains (indicated on the left) without Cre induction and in *TbRAP1*^F2H-NLS-ΔMybL/+^ TRFi cells. IP experiments were done using the 12CA5 HA antibody and IgG (as a negative control). To analyze the IP products, WT *Tb*RAP1 and F2H-*Tb*RAP1ΔMyb were detected with an anti-*Tb*RAP1 rabbit antibody (16), and other F2H-tagged *Tb*RAP1 mutants were detected by the HA probe antibody in Western blotting. To differentiate *Tb*RAP1 and F2H-*Tb*RAP1ΔMyb, proteins were separated on a 7.5% Tris polyacrylamide gel for 7 h 40 min.

To test whether *Tb*RAP1 has any self-interaction ability, we performed co-IP experiments in cells expressing an F2H-*Tb*RAP1 from one of its endogenous alleles. Since *Tb*RAP1 interacts with *Tb*TRF, these co-IP experiments were performed in *Tb*TRF RNAi cells. Both before and after depletion of *Tb*TRF by RNAi (Fig. 7A, right), we detected WT *Tb*RAP1 in the IP product when IP was performed using the 12CA5 HA antibody (Fig. 7A, left). Therefore, *Tb*RAP1 interacts with itself, and this interaction is independent of *Tb*TRF.

To further examine which domain of *Tb*RAP1 is required for its self-interaction, we tested whether any F2H-tagged *Tb*RAP1 domain deletion mutants showed co-IP with the WT *Tb*RAP1. In *TbRAP1*^F/F2H-mut^ cells (without Cre induction) and in *TbRAP1*^F2H-NLS-ΔMybL/+^ TRFi cells, IP experiments were performed using the 12CA5 HA antibody, and the IP products were examined by Western blotting using both the HA probe antibody (Fig. 7B, right) and the rabbit *Tb*RAP1 antibody (16) (Fig. 7B, left). In all cells except *TbRAP1*^F/F2H-ΔBRCT^, WT *Tb*RAP1 was detected in the IP products (Fig. 7B, left), indicating that BRCT is essential for *Tb*RAP1 self-interaction. Although F2H-*Tb*RAP1ΔBRCT was expressed at a lower-than-WT level, it was expressed at a higher level than F2H-*Tb*RAP1ΔNT and *Tb*RAP1ΔMybLΔRCT-F2H-NLS_r_ (Fig. 3B), while the latter two mutants interacted with the WT protein (Fig. 7B, left). Therefore, the lack of interaction between F2H-*Tb*RAP1ΔBRCT and WT *Tb*RAP1 is unlikely to have been due to the low level of expression of the mutant.

## DISCUSSION

RAP1 orthologs are conserved from protozoa to mammals (4–16), and they have similar domain structures (4, 12, 16, 65) and essential telomeric and nontelomeric functions (96). *Tb*RAP1 also has the BRCT, Myb, MybLike, and RCT functional domains, like other RAP1 orthologs (16). *Tb*RAP1 is essential for VSG silencing and telomere/subtelomere integrity and stability (16, 44, 88). However, whether *Tb*RAP1’s domains are required for these functions was unknown. Study of *Tb*RAP1 domain functions was partly limited by the fact that *Tb*RAP1 is essential for cell proliferation (16). Previous studies of *Tb*RAP1 functions were heavily dependent on the use of conditional RNAi to deplete *Tb*RAP1 (16, 44, 88). Although expressing double-stranded RNA (dsRNA) of the *TbRAP1* full-length gene (16, 88), the BRCT fragment, or the RCT fragment (44) can efficiently deplete *Tb*RAP1, expressing dsRNA of the 3′ untranslated region (3′UTR) of *Tb*RAP1 cannot. Therefore, the RNAi approach is not suitable for studying phenotypes of all domain deletion mutants or point mutations of *Tb*RAP1. In this study, we took advantage of the Cre-loxP system (91) and established a series of strains in which one endogenous *TbRAP1* allele is flanked by two repeats of loxP, allowing its conditional deletion upon Cre induction. We confirmed that this conditional deletion was able to efficiently deplete the *Tb*RAP1 protein and mRNA. Most importantly, we are now able to examine the phenotypes of a series of *Tb*RAP1 mutants that lack individual functional domains or carry point mutations, even if the mutants are lethal.

In this study, we found that none of the domain deletion mutants of *Tb*RAP1 could support normal cell growth. F2H-*Tb*RAP1ΔNT, F2H-*Tb*RAP1ΔBRCT, and *Tb*RAP1ΔMybLΔRCT-F2H-NLS_r_ were expressed at much lower levels than the WT *Tb*RAP1, while F2H-*Tb*RAP1ΔMyb, F2H-*Tb*RAP1ΔMybL, and F2H-NLS-*Tb*RAP1ΔMybL were expressed at the same level as WT *Tb*RAP1. Therefore, the N terminus, BRCT, and RCT are required for normal *Tb*RAP1 protein level, and the low level of protein expression is most likely the reason why these mutants do not support normal cell growth. F2H-*Tb*RAP1ΔMyb and F2H-NLS-*Tb*RAP1ΔMybL were localized in the nucleus and expressed at the same level as the WT protein, but these mutants still had a severe growth defect, indicating that Myb and MybLike domains are essential for normal cell growth. Only the DNA binding domains of *Sc*RAP1 (Myb and MybLike) are essential for cell viability, and its RCT domain, which is important for telomere length regulation and telomeric silencing, is not essential for cell survival (66). This leads us to speculate that the *Tb*RAP1 Myb and/or MybLike domains may have DNA binding activities. Although Myb domains are frequently involved in DNA binding (97), the human RAP1 Myb domain does not seem to have any DNA binding activity due to its negative surface charge on the third helix, which is typically involved in DNA recognition (70). In addition, the *Sc*RAP1 MybLike domain was revealed to fold into a DNA binding motif only after its crystal structure was solved (69). It will be interesting to investigate whether *Tb*RAP1 Myb and MybLike domains have any DNA binding activities, but further protein structural analysis may be necessary.

Myb domains can also mediate protein-protein interactions (98, 99). We found that *Tb*RAP1 Myb interacts with *Tb*TRF, providing another piece of evidence that the Myb domain can have an important function in protein-protein interaction. Nevertheless, whether the interaction between *Tb*RAP1 and *Tb*TRF is essential for cell survival and/or VSG silencing is still unknown. Further investigation will be necessary to identify key residues in *Tb*RAP1 Myb that are critical for *Tb*TRF interaction, which will help address this issue. Human RAP1 uses its C-terminal RCT domain to interact with TRF2’s linker region (4, 68). Similarly, Schizosaccharomyces pombe RAP1 also uses its C-terminal RCT domain to interact with Taz1, a functional homologue of mammalian TRF1/2 (65, 68), indicating that this interaction interface is conserved from yeast to mammals. However, we found that the *Tb*RAP1 Myb domain is critical for *Tb*TRF interaction. It is interesting that the RAP1-TRF interaction is preserved from kinetoplastids to mammals and yet the functional domains that accomplish this goal have changed.

The transcription profile of more than 10,000 genes was found to have changed in *TbRAP1*^F/F2H-ΔMyb^ cells after induction of Cre. In particular, ∼2,700 *VSG* genes were upregulated. Since the VSGnome identified more than 2,500 *VSG* genes and pseudogenes in the Lister 427 genome (72), this means nearly all of the *VSG* genes were upregulated, further validating that *Tb*RAP1 has an essential function in silencing *VSG* genes and that Myb is essential for this function. Interestingly, RNA-seq also identified nearly 3,000 genes that are downregulated in *TbRAP1*^F/F2H-ΔMyb^ cells, although the fold change in mRNA levels was much lower than that seen with the upregulated genes. *Sc*RAP1 has been well known for both its transcription activation and its repression functions (50), and *Sc*RAP1 is required for ribosomal protein gene activation (8). Our observation suggests that *Tb*RAP1 may have functions similar to those of *Sc*RAP1, although *Tb*RAP1’s transcription activation effect appears to be much weaker than its repressive effect. Some of the downregulated genes encode ribosomal proteins, suggesting that *Tb*RAP1 may also participate in ribosomal protein gene activation. However, further validation is necessary to confirm *Tb*RAP1’s role as a transcription activator.

Importing >45-kDa nuclear proteins frequently depends on the presence of NLS, which can be recognized by importin α/β proteins (100). After the importin-cargo complex is transported through the nuclear pore complexes, binding of RanGTP to the importin β dissociates the complex to release cargo into the nucleoplasm (100). However, nuclear proteins can also be transported into the nucleus through NLS-independent mechanisms, such as interacting with a protein partner that contains a NLS (101). *Tb*RAP1 is a nuclear protein (16). However, how *Tb*RAP1 is imported into the nucleus was unknown. Here, we show that importin α directly interacts with *Tb*RAP1 and that this interaction depends on the MybLike domain, which contains a predicted bipartite NLS. Additionally, F2H-*Tb*RAP1ΔMybL is not localized in the nucleus, while *Tb*RAP1ΔMybLΔRCT-F2H-NLS_r_ with the second half of the *Tb*RAP1 NLS, is. Therefore, *Tb*RAP1’s nuclear localization depends on the NLS in the MybLike domain and requires its recognition by importin α. Addition of an SV40 large T NLS to the N terminus of *Tb*RAP1ΔMybL can target the mutant to the nucleus, indicating that the NLS and importin α are well conserved with other known classical NLS and importin α proteins, respectively. Interestingly, F2H-NLS-*Tb*RAP1ΔMybL still interacts with *Tb*TRF. However, without any NLS, F2H-*Tb*RAP1ΔMybL is localized in the cytoplasm, indicating that the interaction between *Tb*RAP1 and *Tb*TRF is not sufficient to bring *Tb*RAP1 into the nucleus.

Human RAP1 interacts with itself through the RCT domain (4), although the function of this self-interaction is unknown. Human RAP1 interacts tightly with TRF2 with equal stoichiometry (102), and TRF2 homodimerizes (103). It is possible that homodimerization of human RAP1 allows a better interaction with TRF2. The *Tb*TRF-*Tb*RAP1 interaction is much weaker than the *Tb*TRF-*Tb*TIF2 interaction (4, 86), and whether *Tb*TRF interacts with *Tb*RAP1 with equal stoichiometry is unknown. Therefore, whether *Tb*RAP1 self-interaction contributes to its interaction with *Tb*TRF is not clear. We found that *Tb*RAP1’s self-interaction is independent of *Tb*TRF, indicating that this self-interaction is direct and not mediated by the *Tb*RAP1-*Tb*TRF interaction. Additionally, *Tb*RAP1 self-interaction and *Tb*RAP1-*Tb*TRF interaction require different functional domains. The BRCT domain is also important for a normal level of *Tb*RAP1 protein. It is possible that *Tb*RAP1 is better stabilized with self-interaction, possibly by preventing *Tb*RAP1 degradation by proteases. Further analysis with point mutations in the BRCT domain that specifically abolish *Tb*RAP1 self-interaction will be useful to reveal its function and help understand the mechanism of protein stabilization.

The fact that the functional interactions of RAP1 with other telomere proteins, such as TRF, or with itself are conserved from kinetoplastids to mammals suggests that these functions of RAP1 orthologs are critical for essential cellular processes. However, throughout evolution, different organisms have used different approaches to achieve the same goal. Hence, the detailed protein-protein interaction interfaces have been changed even though the consequential protein complex is still preserved. We have observed a similar scenario in interactions of *Tb*TRF and *Tb*TIF2 (86). Although *Tb*TRF interacts with *Tb*TIF2 (86) and mammalian TRF1 and TRF2 interact with TIN2 (104, 105), the two protein pairs interact with different interfaces (86). Therefore, conserved protein-protein interactions without a conserved interaction interface can be common among many telomere protein homologues.

*Tb*RAP1, as with its orthologs in other organisms, has multiple protein-protein interaction domains. A common theme appears to pertain for most RAP1 orthologs: RAP1 interacts with different protein partners to perform different cellular tasks (50). Our results further suggest that *Tb*RAP1 interacts with different partners through different functional domains to achieve various goals. With our established Cre-loxP system, we will be able to investigate details of the functions of each *Tb*RAP1 domain in the future.

## MATERIALS AND METHODS

### T. brucei strains and plasmids.

All T. brucei strains used in this study (listed in Table S1 in the supplemental material) were derived from bloodstream-form Lister 427 cells that express VSG2 as well as a T7 polymerase and the Tet repressor (also known as the single marker or SM strain) (106). All BF T. brucei cells were cultured in HMI-9 medium supplemented with 10% fetal bovine serum (FBS) and appropriate antibiotics.

To establish the *TbRAP1*^F/+^ strain, the NotI/XhoI-digested *Tb*RAP1-5′UTR-loxP-*HYGGFPTK* plasmid and NotI/XhoI-digested *Tb*RAP1-3′UTR-*BSDGFPTK*-loxP plasmid were sequentially transfected into SM cells. The A1 and A3 clones of *TbRAP1*^F/+^ (-Cre-EP1) were confirmed by Western and Southern analyses (see Fig. S1A and B in the supplemental material). The conditional Cre-expressing Cre-EP1 plasmid with the phleomycin resistance (*BLE*) marker (91) was subsequently inserted into an rDNA spacer region to generate the final *TbRAP1*^F/+^ strain. Clones A1 and B1 were verified by their sensitivity to hygromycin and blasticidin after induction of Cre expression by the use of doxycycline (Fig. S1C).

To establish the *TbRAP1*^F/−^ strain, the NotI/XhoI-digested pSK-*Tb*RAP1-ko-*PUR* plasmid was transfected into *TbRAP1*^F/+^ cells. The pSK-*Tb*RAP1-ko-*PUR* plasmid contains *PUR* flanked by sequences upstream and downstream of the *Tb*RAP1 open reading frame (ORF), respectively.

All *TbRAP1*^F/F2H-mut^ strains were established using the same strategy. N-terminal F2H-tagged *Tb*RAP1ΔNT, *Tb*RAP1ΔBRCT, *Tb*RAP1ΔMyb, *Tb*RAP1ΔMybL, and NLS-*Tb*RAP1ΔMybL mutants flanked by sequences upstream and downstream of the *TbRAP1* gene, together with a *PUR* marker, were cloned into pSK to generate respective targeting constructs. The *Tb*RAP1ΔMybLΔRCT-F2H-NLS_r_ mutant flanked by sequences upstream and downstream of the *TbRAP1* gene was also cloned into pSK with a *PUR* marker to generate the mutant targeting construct. All mutant targeting plasmids were digested with SacII (or with PvuII in the case of ΔMybLΔRCT) before transfection of the *TbRAP1*^F/+^ cells was performed to generate the corresponding *TbRAP1*^F/F2H-mut^ strains. All mutant strains were confirmed by Southern analyses.

For examination of *Tb*RAP1 self-interaction in the presence and absence of *Tb*TRF, a *Tb*TRF RNAi (TRFi) strain was first established by transfection of the NotI-digested pZJMβ-*Tb*TRF-Mid1 RNAi construct (89) into SM cells. Subsequently, one endogenous *TbRAP1* allele was tagged with an N-terminal F2H tag by transfection of a SacII-digested pSK-*PUR*-F2H-*Tb*RAP1-tar2 construct into the TRFi cells.

Insertion of 13 C-terminal repeats of myc into one endogenous allele of importin α was performed in *TbRAP1*^+/+^, *TbRAP1*^F2H+/+^ TRFi, and *TbRAP1*^F2H-ΔMybL/+^ TRFi cells. *TbRAP1*^F2H-ΔMybL/+^ TRFi was obtained by replacing one endogenous *TbRAP1* allele with the F2H-*Tb*RAP1ΔMybL mutant in TRFi cells as described above. (The *TbRAP1*^F2H-NLS-ΔMybL/+^ TRFi strain used for *Tb*RAP1 co-IP analysis was established in a similar way.) The importin α C-terminal myc_13_ tagging fragment was amplified by PCR with primers OBL-TbIMPORT-CT-MYC-FW (5′-GAGGGGGCACCTCAGCAGTTTGAGTTGGGGATGGATCATGGAGATCCAAATGGACAGCCACCACAGGGCCAGTTCGATCTTATCCCCGGGTTAATTAACG-3′) and OBL-TbIMPORT-CT-MYC-BW (5′-GTATATATATATACACGTGCCCTCCTTCTTCACCATTTTCATCCCTTTCATCTCGATCTCATTATATTTATCAACTTTTCTCATCTAGATTCCTTTGCCC-3′) by the use of a plasmid containing myc_13_ and a *HYG* marker as the template. The PCR product was then purified and transfected into *TbRAP1*^+/+^, *TbRAP1*^F2H+/+^ TRFi, and *TbRAP1*^F2H-ΔMybL/+^ TRFi cells.

### Coimmunoprecipitation.

A total of 200 million T. brucei cells in log-phase growth were used for each IP using appropriate antibody or IgG (as a negative control). IP products were pulled down by the use of Dynabeads protein G (Life Technologies) and split equally for two Western blotting analyses using appropriate antibodies. A 1% volume of input sample was loaded as a control. Since F2H-*Tb*RAP1ΔNT, F2H-*Tb*RAP1ΔBRCT, and *Tb*RAP1ΔMybLΔRCT-F2H-NLS_r_ express at low levels, 500 million mutant cells were used for each IP. A 1% volume of input was loaded in Western analysis as a control.

### Immunofluorescence analyses.

IF experiments were performed as described previously (86). Specifically, cells were fixed with 2% formaldehyde at room temperature for 10 min, permeabilized in 0.2% NP-40–1× phosphate-buffered saline (PBS) at room temperature for 5 min and blocked by the use of 1× PBS–0.2% cold fish gelatin–0.5% bovine serum albumin (BSA) at room temperature twice for 10 min each time, followed by incubation with the primary antibody at room temperature for 2 h and the secondary antibody at room temperature for 1 h. Cells were then washed with 1× PBS–0.2% cold fish gelatin–0.5% BSA and 1× PBS followed by staining with 0.5 μg/ml DAPI (4′,6-diamidino-2-phenylindole) and by mounting of coverslips on slides. Images were taken by a DeltaVision Elite deconvolution microscope. Images were deconvolved using SoftWoRx.

### T. brucei cDNA library.

A normalized T. brucei cDNA library was prepared by Bio S&T. Briefly, using a modified SMART cDNA synthesis method, the total RNA from WT T. brucei cells was used for synthesis of cDNA with either oligo(dT) or a random primer. The cDNA was normalized and amplified, after which it was inserted into a modified pGAD T7 yeast expression vector.

### Yeast 2-hybrid screen.

The *Tb*RAP1 MybLike domain was cloned into the pBTM116 vector and transformed into the yeast strain L41 [*MAT*α *his 3D200 trp1-901 leu2-3*,*112 ade2 LYS*::(*lexAop*)*4-HIS3 URA3*::(*lexAop*)*8–lacZ gal4 gal80*]. The resulting cells were transformed with the normalized T. brucei cDNA library. A total of 16.5 million primary transformants were plated onto synthetic drop-out (SD) plates without tryptophan, leucine, or histidine. A total of 711 clones were obtained from this initial screening. Subsequently, these clones were tested by the use of filter lift assays, and 508 candidates were verified to express the reporter *lacZ* gene. The pGAD T7 candidate plasmids were isolated from these yeast transformants and T. brucei gene insertions were analyzed by restriction digestion, PCR, and sequencing.

### RNA-seq.

Cre expression was induced by the use of doxycycline in *TbRAP1*^F/F2H-ΔMyb^ and *TbRAP1*^F/+^ cells for 30 h, after which total RNA was isolated and purified through RNeasy columns (Qiagen). Three independent inductions were performed as biological replicates. RNA samples were run on a BioAnalyzer 2100 system (Agilent Technologies) using an Agilent RNA 6000 Nano kit to verify the RNA quality and then sent to Novogene for library preparation and RNA high-throughput sequencing.

The following processes were performed at Novogene.

### (i) RNA quantification and qualification.

RNA degradation and contamination were monitored on 1% agarose gels. RNA purity was checked using a NanoPhotometer spectrophotometer (Implen). RNA integrity and quantitation were assessed using an RNA Nano 6000 assay kit on a Bioanalyzer 2100 system (Agilent Technologies).

### (ii) Library preparation for transcriptome sequencing.

Sequencing libraries were generated using a NEBNext Ultra RNA library prep kit for Illumina (NEB, USA) using 1 μg poly(A) RNA according to the manufacturer’s protocol, and index codes were added to attribute sequences to each sample. Briefly, mRNA was purified from total RNA using poly(T) oligonucleotide-attached magnetic beads. Fragmentation was carried out using divalent cations under conditions of elevated temperature in NEBNext first-strand synthesis reaction buffer (5×). First-strand cDNA was synthesized using random hexamer primers and Moloney murine leukemia virus (M-MuLV) reverse transcriptase (RNase H-). Second-strand cDNA synthesis was performed using DNA polymerase I and RNase H. The remaining overhangs were converted into blunt ends via exonuclease/polymerase activities. After adenylation of 3′ ends of DNA fragments, NEBNext Adaptor with a hairpin loop structure was ligated to prepare for hybridization. In order to select cDNA fragments preferentially of length 150 to 200 bp, the library fragments were purified with an AMPure XP system (Beckman Coulter). A 3-μl volume of USER Enzyme (NEB, USA) was used with size-selected, adaptor-ligated cDNA at 37°C for 15 min followed by 5 min at 95°C followed by PCR, which was performed with Phusion high-fidelity DNA polymerase, universal PCR primers, and an Index (X) primer. PCR products were purified (AMPure XP system), and library quality was assessed on an Agilent Bioanalyzer 2100 system.

### (iii) Clustering and sequencing (Novogene Experimental Department).

Clustering of the index-coded samples was performed on a cBot cluster generation system using a cBot-HiSeq (HS) paired-end (PE) cluster kit (Illumina) according to the manufacturer’s instructions. After cluster generation, the library preparations were sequenced on an Illumina platform and 125-bp and 150-bp paired-end reads were generated.

### RNA-seq data analysis.

The RNA-seq data were analyzed by Novogene as follows.

### (i) Quality control.

Raw reads of fastq format were first processed through the use of Novogene perl scripts. In this step, clean reads were obtained by removing reads containing adapters, reads containing poly(N), and low-quality reads. At the same time, the levels of Q20, Q30, and GC content of the clean reads were calculated. All downstream analyses were performed on the basis of the clean reads with high quality.

### (ii) Mapping of reads to the reference genome.

The T. brucei Lister 427 genome TriTrypDB-45_TbruceiLister427_2018_Genome.fasta and its annotation TriTrypDB-45_TbruceiLister427_2018.gff were downloaded from TriTrypDB and used as the references. The index of the reference genome was built using hisat2 2.1.0, and paired-end clean reads were aligned to the reference genome using HISAT2.

### (iii) Quantification of gene expression levels.

HTSeq v0.6.1 was used to calculate the number of reads mapped to each gene. The number of fragments per kilobase per million (FPKM) was calculated for each gene on the basis of the length of the gene and the number of reads mapped to the gene.

### (iv) Differential expression analysis.

Differential expression analysis of two conditions/group (three biological replicates per condition) was performed using the DESeq R package (1.18.0). The DESeq R package provides statistical routines for determining differential expression in digital gene expression data using a model based on the negative binomial distribution. The resulting *P* value*s* were adjusted using the Benjamini and Hochberg approach for controlling the false-discovery rate. Genes with an adjusted *P* value of <0.05 found by DESeq were assigned as differentially expressed.

### Data availability.

The RNA-seq data set determined in this work has been submitted to NCBI GEO under accession number GSE143456.
