# Supplementary material for: Trypanosoma brucei RAP1 Has Essential Functional Domains That Are Required for Different Protein Interactions
Source: mSphere. 2020 Feb 26;5(1):e00027-20. doi: 10.1128/mSphere.00027-20 (PMC7045384; doi:10.1128/mSphere.00027-20)
Supplement: TABLE S1 [file mSphere.00027-20-st001.docx]

Supplemental Table 1

| Strain | *Tb*RAP1 alleles | | Calculated MW (kD) of *Tb*RAP1 expressed from the 2nd allele* | *Tb*TRF alleles | | *Tb*TRF RNAi |
| --- | --- | --- | --- | --- | --- | --- |
|  | allele one | allele two |  | allele one | allele two |  |
| *TbRAP1*^+/+^ | WT | WT | 92.83 | WT | WT | No |
| *TbRAP1*^F/+^ | floxed | WT | 92.83 | WT | WT | No |
| *TbRAP1*^F/-^ | floxed | replaced by *PUR* | 0 | WT | WT | No |
| *TbRAP1*^F/F2H+^ | floxed | F2H-*Tb*RAP1 | 97.22 | WT | WT | No |
| *TbRAP1*^F/F2H-∆NT^ | floxed | F2H-*Tb*RAP1∆NT | 76.17 | WT | WT | No |
| *TbRAP1*^F/F2H-∆BRCT^ | floxed | F2H-*Tb*RAP1∆BRCT | 76.25 | WT | WT | No |
| *TbRAP1*^F/F2H-∆Myb^ | floxed | F2H-*Tb*RAP1∆Myb | 88.77 | WT | WT | No |
| *TbRAP1*^F/F2H-∆MybL^ | floxed | F2H-*Tb*RAP1∆MybL | 83.22 | WT | WT | No |
| *TbRAP1*^F/F2H-NLS-∆MybL^ | floxed | F2H-NLS-*Tb*RAP1∆MybL | 85.44 | WT | WT | No |
| *TbRAP1*^F/∆MybL∆RCT-F2H-NLSr^ | floxed | *Tb*RAP1∆MybL∆RCT-F2H-NLSr | 59.73 | WT | WT | No |
| *TbRAP1*^-/F2H+^ | replaced by *HYG* | F2H-*Tb*RAP1 | 97.22 | WT | WT | No |
| *TbRAP1*^F2H+/+^ TRFi | WT | F2H-*Tb*RAP1 | 97.22 | WT | WT | Yes |
| *TbRAP1*^F2H-∆MybL/+^ TRFi | WT | F2H-*Tb*RAP1∆MybL | 83.22 | WT | WT | Yes |
| *TbRAP1*^F2H-NLS-∆MybL/+^ TRFi | WT | F2H-NLS-*Tb*RAP1∆MybL | 85.44 | WT | WT | Yes |

* TbRAP1 always runs slower than expected in polyacrylamide gels.
