# Supplementary material for: Trypanosoma brucei RAP1 Has Essential Functional Domains That Are Required for Different Protein Interactions
Source: mSphere. 2020 Feb 26;5(1):e00027-20. doi: 10.1128/mSphere.00027-20 (PMC7045384; doi:10.1128/mSphere.00027-20)

Afrin et al. Figure S3

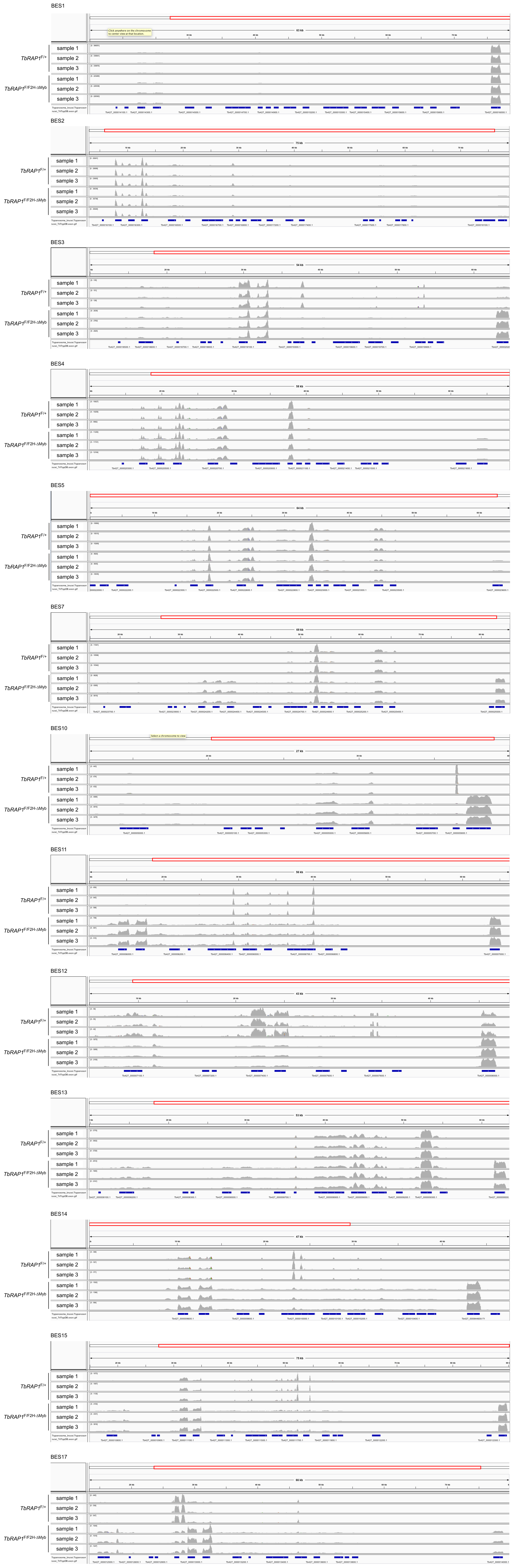

Supplement: FIG S3 [file mSphere.00027-20-sf003.pdf]
